# What Is the Fuss about Integrins and the Tumor Microenvironment?

**DOI:** 10.3390/cancers11091296

**Published:** 2019-09-03

**Authors:** Johannes A. Eble, Donald Gullberg

**Affiliations:** 1 Institute of Physiological Chemistry and Pathobiochemistry, University of Münster, Waldeyerstr. 15, 48149 Münster, Germany; 2 Department of Biomedicine and Centre for Cancer Biomarkers, University of Bergen, Jonas Lies vei 91, NO-5009 Bergen, Norway

In recent years the tumor microenvironment (TME) has received increasing attention. 

A major cell type in the TME is an ill-defined cell type often called cancer-associated fibroblast (CAF), which influences tumor growth, formation of stem cell niches, immunosuppression, metastasis and chemoresistance [1,2]. 

With new technologies, we currently see a burst in activity aiming at demystifying fibroblast and CAF heterogeneity in the tumor stroma. A number of CAF subtypes have been defined within tumor stroma. Pioneer work has defined two major types of fibroblasts in pancreatic cancer, inflammatory CAFs and myofibroblastic CAFs [3], and four major subclasses of CAFs in breast cancer distinguished by different levels of αSMA and fibroblasts activation protein (FAP) expression [4,5]. Due to their plasticity and dynamic nature it has been suggested that the CAF subtypes do not represent fixed cell types, but rather represent fibroblast “states” [6]. However, epigenetic changes do result in more stable CAF phenotypes [7,8]. Indirect evidence suggests that some subpopulations of CAFs are tumor-supportive whereas others are tumor-suppressive [9,10]. Major challenges in all forms of tumor fibrosis include characterizing the degree of fibroblast heterogeneity, defining the origin of pro-fibrotic cells (also the potential targets of anti-fibrosis therapy), and characterizing the dynamics of different biomarkers, which can be used to follow the fibrotic process as well as serve as potential therapeutic targets.

Apart from cellular components, the TME also encompasses the extracellular space, which contains both soluble cytokines and insoluble extracellular matrix (ECM) components. The latter tend to assemble into supramolecular structures, which serve as scaffold for the cells. The ECM varies not only in its biochemical components but is also characterized by its biophysical state, such as rigidity or tension, which the scaffold has to bear. Moreover, the ECM is a medium, via which cells may communicate, allowing mechanical forces and tensions to be transmitted between different cells within the tumor stroma. To this end, cells within the TME, like in normal tissue, have to come in physical contact with the ECM. Integrins are a principal class of cell adhesion molecules that serve as ECM receptors [11], which not only convey mechanical forces but also signals between the ECM and the cytoskeleton. In this fundamental property they also play highly relevant roles in the communication between the different cell types and the ECM in the TME. In this special issue of *Cancers*, we will focus on the role of integrins in relation to this important compartment of the tumor and discuss different aspects of TME-mediated effects on tumor progression, metastasis and chemoresistance. We have tried to collect an exciting mix of articles which deal with tumor TME and CAFs.

Studies of the role of integrins in relation to tumor cell–CAF interactions can mean two things: either studies of integrins on CAFs or studies of integrins on tumor cells which can affect CAF function or be affected by CAFs. The approach of trying to use integrins as therapeutic targets in cancer is complex. Some of this complexity is due to the widespread expression of many integrins, making targeting and interpretation of results difficult. New hope has recently been injected into the field with the focus on either (1) integrins involved in activating Transforming growth factor (TGF)-β or (2) integrins having a restricted tissue distribution within the tumor. One avenue of research preferentially utilizes integrin inhibitors such as antibodies as vehicles to deliver drugs to the tumor. Alternatively, another strategy is to develop small molecules that can be used either directly as integrin inhibitors or in the context of drug delivery. Integrin α10β1 is an integrin which shows a very restricted tissue distribution. In normal tissues its expression is limited to chondrocytes and mesenchymal stem cells, but it has now been found to also be upregulated on glioblastoma cells, and constitutes a new target for this tumor type [12]. The unexpected and surprising upregulation of a cartilage molecule in this tumor type demonstrates how unpredictable integrin science can be and encourages further studies of tissue-restricted integrins. Integrin α11β1 is another collagen-binding integrin with a restricted tissue distribution largely confined to subsets of fibroblasts. In this issue, Zeltz et al. demonstrated that it is expressed on CAFs in multiple solid tumor types [13] and that it regulates LOXL1 in non-small cell lung cancer [14]. In contrast, the laminin-binding integrin α3β1 is abundantly expressed in several cells both of normal and tumor tissue. However, Martins Cavaco et al. show in this issue that this integrin seems to be not only an expression marker for CAFs but also is indispensable driver of differentiation of fibroblasts into CAFs within the TME of pancreatic duct adenocarcinomas [15]. Laminin-332, an ECM protein ectopically expressed in the tumor stroma, is a ligand for α3β1 integrin and supports the α3β1 integrin-mediated transition of tumor-supporting fibroblasts. DiPersio et al. brings more examples of the varying roles of integrins on both CAFs and tumor cells in their review [16]. In more translational approaches, two publications of this special issue on integrins in cancer highlight compounds that target integrins on cancer cells [17] or an immune check-point receptor along with integrins [18], resulting in tumor cell elimination. Extending the most recent advancement of chimeric antigen receptor (CAR) T-cells, Whilding et al. observed an experimental tumor regression by using CAR T-cells expressing Il-8 receptors which attack tumor cell-expressed αVβ6 integrin [19]. The IL-8 cytokine is upregulated in several tumor entities and attracts CAR T-cells to the TME. Additional cell types are resident or immigrate into tumor tissue. All of them have different sets of integrins. Among them, the different αV integrins were proposed as targetable receptors. Their potential in anti-tumor therapy is reviewed by Alday-Parejo et al. [20] and Brown and Marshall [21].

Integrins not only mediate cell contacts between cells and ECM, but they also associate with ECM-degrading proteases. This ‘unholy alliance’ of ECM binding and cleavage is typical for invadosomes, cell protrusions typically found in tumor cells, and is highlighted by the work of Peláez et al. [22]. The CAFs also secrete matrix metalloproteinases in a differentiation-dependent manner and thus support the ECM remodeling or degradation in favor of the invading tumor cells. This aspect is highlighted by Eiro et al. [23] in this issue.

Both the composition and the biophysical state of the ECM are essential parameters of the TME. Mechanical forces exerted by CAFs set the ECM under tension, a condition which cancer cells sense via integrins and determines the cancer cell progression [24]. Moreover, these integrin-mediated signals of mechanical forces also influence the cellular signals triggered by growth factor receptors on both tumor cells and CAFs, thereby underlining the importance of several components within the TME [25]. 

In summary, this Special Issue of *Cancers* is a collection of articles discussing the role of integrins in cancer with a special reference to cancer-associated fibroblasts within the tumor microenvironment. Since the function of CAFs in the TME is very tightly connected with the functions of the other cell types present, the interconnectivity of CAFs always needs to be taken into consideration. In the coming years, more work is needed to advance the field with regard to the role of CAF integrins and their therapeutic potential in anti-stroma therapy, chemoresistance and immunotherapy. 
